# First experience with MR-guided focused ultrasound in the treatment of Parkinson's disease

**DOI:** 10.1186/2050-5736-2-11

**Published:** 2014-05-31

**Authors:** Anouk Magara, Robert Bühler, David Moser, Milek Kowalski, Payam Pourtehrani, Daniel Jeanmonod

**Affiliations:** 1 Praxis für Neurologie, Monbijoustrasse 73, 3007 Bern, Switzerland; 2 Neurological Division, Bürgerspital Solothurn, Schöngrünstrasse 38, 4500 Solothurn, Switzerland; 3 Sonimodul, Center for Ultrasound Functional Neurosurgery, Leopoldstrassse 1, CH-4500 Solothurn, Switzerland; 4 Privatklinik Obach, Leopoldstrasse 5, 4500 Solothurn, Switzerland; 5 Rodiag Diagnostics Centers, Leopoldstrasse 3, 4500 Solothurn, Switzerland

## Abstract

**Background:**

Radiofrequency (RF) subthalamotomies have been proposed since the 1960s to treat patients suffering from Parkinson's disease (PD). Recently, the magnetic resonance (MR)-guided focused ultrasound technology (MRgFUS) offers the possibility to perform subthalamic thermocoagulations with reduced risks and optimized accuracy. We describe here the initial results of the MRgFUS pallidothalamic tractotomy (PTT), an anatomical and physiological update of the earlier subthalamotomies.

**Methods:**

Thirteen consecutive patients suffering from chronic (mean disease duration 9.7 years) and therapy-resistant PD were treated unilaterally with an MRgFUS PTT. Primary relief assessment indicators were the score reduction of the Unified Parkinson Disease Rating Scale (UPDRS) and the patient estimation of global symptom relief (GSR) taken at 3 months follow-up. Final temperatures at target were between 52°C and 59°C. The MR examinations were performed before the treatment, 2 days and 3 months after it. The accuracy of the targeting was calculated on 2 days post-treatment MR pictures for each PTT lesion.

**Results:**

The first four patients received a PTT using the lesional parameters applied for thalamotomies. They experienced clear-cut recurrences at 3 months (mean UPDRS relief 7.6%, mean GSR 22.5%), and their MR showed no sign of thermal lesion in T2-weighted (T2w) images. As a consequence, the treatment protocol was adapted for the following nine patients by applying repetition of the final temperatures 4 to 5 times. That produced thermocoagulations of larger volumes (172 mm^3^ against 83 mm^3^ for the first four patients), which remained visible at 3 months on T2w images. These nine patients enjoyed a mean UPDRS reduction of 60.9% and a GSR of 56.7%, very close to the results obtained with radiofrequency lesioning. The targeting accuracy for the whole patient group was 0.5, 0.5, and 0.6 mm for the anteroposterior (AP), mediolateral (ML), and dorsoventral (DV) dimensions, respectively.

**Conclusions:**

This study demonstrated the feasibility, safety, and accuracy of the MRgFUS PTT. To obtain similar results as the ones of RF PTT, it was necessary to integrate the fact that white matter, in this case, the pallidothalamic tract, requires repeated thermal exposition to achieve full lesioning and thus full therapeutic effect.

## Background

Pharmacological therapy of Parkinson's disease (PD), based on l-dopa and dopamine agonists, is well established, specific, and effective. However, therapy resistance, fluctuations, and dyskinesias may develop over time. To provide treatment to these chronically and severely affected patients, surgical approaches have been developed since the 1950s and 1960s. They comprise radiofrequency lesion and high-frequency stimulation (HFS) at the level of the motor thalamus, subthalamic nucleus, or the internal part of the globus pallidus (GPi), HFS of the subthalamic nucleus being mostly used since a few years [1-6].

In the 1960s, radiofrequency lesioning in the subthalamus was proposed and explored by different authors and provided similarly to interventions in the GPi, symptom relief for the whole PD triad [7-9]. Two other elements lead us to update this approach [10,11]: firstly, the evidence of a state of thalamic overinhibition followed by thalamocortical overactivity [10,12,13], and secondly the anatomical evidence for a funneling of a large majority of pallidothalamic fibers in the field H1 of Forel (or fasciculus thalamicus). We performed a detailed histological topographical mapping of the fiber tracts in the subthalamus [14] to allow a precise targeting of the fasciculus thalamicus and proposed the term ‘pallidothalamic tractotomy’ (PTT) to indicate the targeted subthalamic structure, i.e., the pallidothalamic tract. Through this tract of around 4-ml diameter, the pallidothalamic fibers of the fasciculus lenticularis (or H2 field of Forel) and of the ansa lenticularis are funneled together into the thalamus [15].

Recently, the new technology of magnetic resonance (MR)-guided focused ultrasound technology (MRgFUS) has allowed performing accurate therapeutic thermocoagulations in the thalamus to treat chronic and therapy-resistant neuropathic pain [16,17]. We have started a study in 2011 applying this technology to the treatment of neuropathic pain, PD, and essential tremor. We report here the initial results of the MRgFUS pallidothalamic tractotomy in the treatment of PD. In addition to the assessment of safety and accuracy of this new technology, we wanted to compare the results of MRgFUS and radiofrequency (RF) thermal lesioning processes. We were lead by initial results to consider the relevance of differential tissue resistance to thermal ablation.

## Methods

### Patient population

Thirteen consecutive patients suffering from chronic and therapy-resistant Parkinson's disease (PD) were enrolled for an MRgFUS treatment in the context of a study approved by the Ethics Committee of Aargau/Solothurn and Swissmedic (Study No. 2010/041). They all signed an informed consent form after having been fully instructed about the treatment, its characteristics, risks, and benefits. The patients were sent by neurologists after they had ascertained the resistance to well-established drug treatment comprising l-dopa and dopamine agonists. Selection criteria for stereotactic treatment were as follows:

1) Idiopathic PD as defined primarily by the presence of tremor at rest, hypobradykinesia, and rigidity.

2) At least one of the two clinically most relevant symptoms, tremor at rest and akinesia, reached an intensity of at least 3/4.

3) Symptoms have resisted to optimal pharmacological treatment including l-dopa and other antiparkinsonian drugs for at least 1 year.

4) Absence of dementia.

5) Strongly diminished quality of life.

The mean age of the patients was 64.5 years (range 37 to 82 years), and the mean disease duration is 9.7 years (range 3 to 27 years). Nine patients suffered from tremulo-akinetic PD forms where tremor dominated, one patient from an akinetic form with on-dyskinesias, and 3 from an akineto-tremulous form where akinesia dominated and with on-dyskinesias (see Table 1). The patients were assessed by complete neurological examinations and the filling of the Unified Parkinson Disease Scale (UPDRS), the Mini-Mental State Test (MMST), and the Hospital Anxiety and Depression Score (HADS). Primary relief assessment indicators were the postoperative reduction of the UPDRS score and the post-operative patient estimation of global symptom relief (GSR in percent). Postoperative follow-up examinations and assessments were performed at 3 months by two neurologists not affiliated with the treating neurosurgical center. Considering limb motricity, the relevant UPDRS scores were assessed on the extremities contralateral to PTT. See Table 1 for patient characteristics.

**Table 1:** Patients' “characteristics”

	**Patient no.**	**Sex**	**Age (years)**	**Disease duration (years)**	**Disease form**
Group 1	1	M	72	27	Akineto-tremulous, bilateral
					On-dyskinetic
	2	F	71	12	Tremor-dominant, left-sided
	3	M	82	11	Tremor-dominant, bilateral
	4	M	69	3	Tremor-dominant, bilateral
Group 2	5	M	39	6	Tremor-dominant, left-sided
	6	M	37	4	Tremor-dominant, right-sided
	7	F	64	9	Tremor-dominant, Bilateral
	8	F	71	8	Tremor-dominant, Bilateral
	9	F	70	14	Akineto-tremulous, bilateral
					On-dyskinetic
	10	M	59	7	Akinetic, bilateral
					On-dyskinetic
	11	F	68	10	Tremulo-akinetic, bilateral
	12	M	68	3	Tremulo-akinetic, bilateral
	13	M	68	12	Akineto-tremulous, bilateral
					On-dyskinetic
Means			64.5	9.7	-

Main pre-operative characteristics of the two groups of patients, including sex, age, symptom duration and disease forms.

### Focused ultrasound procedure

**Figure 1:**
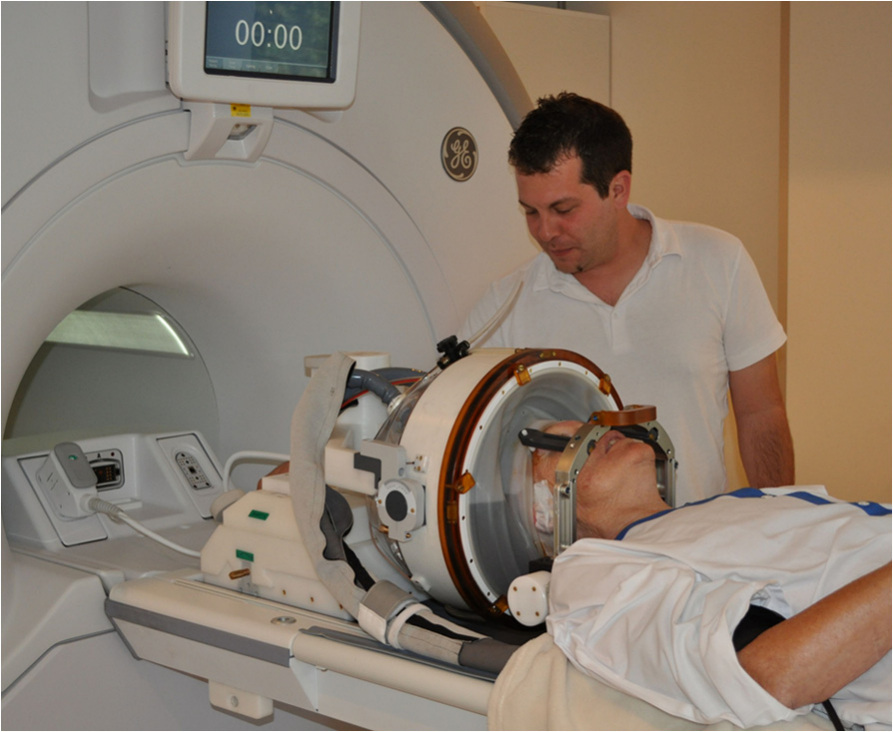
**Intra-operative patient setup.** Patient lies on MR bed with the head immobilized by a metal frame and placed inside the phased-array ultrasound transducer. The space between the head and the transducer has been sealed by a silicone membrane and is being filled with cooled and degassed water.

The treatment was performed in a 3 Tesla (T) MR imaging system (GE Discovery 750, GE Healthcare, Milwaukee, WI, USA) using the ExAblate Neuro device (InSightec, Haifa, Israel), which features a 30-cm-diameter hemispherical 1,024-element phased array transducer operating at 710 kHz and held by a mechanical positioner. Magnetic resonance imaging was performed using the in-built body coil. The patient's scalp was fully and closely shaved, and the head was immobilized by fixation in a Radionics (Integra Radionics, Burlington, VT, USA) MR imaging-compatible frame. A circular flexible silicone membrane with a central hole was stretched around the patient's head and sealed to the outer face of the transducer to contain the degassed and chilled water (15°C–18°C) that was circulated in the volume between the head and the transducer (Figure 1). Axial, sagittal, and coronal T2-weighted fast response, fast spin echo (FRFSE) images were obtained and co-registered to the previously acquired computer tomography (CT) images. The target area, the pallidothalamic tract, was localized on the axial T2-weighted FRFSE images using the stereotactic multiarchitectonic *Morel Atlas of the Human Thalamus and Basal Ganglia*[18]. The coordinates of the target center were at the midcommissural line in the anteroposterior (AP) direction, 7.5 mm lateral to the thalamo-ventricular border in the mediolateral (ML) direction, and 1 mm below to 1 mm above the intercommissural line (according to its length) in the dorsoventral (DV) direction. These coordinates were entered into the planning software of the ExAblate. Several low-power sonications were applied to induce temperatures up to 45°C, known to be below the ablation threshold. They allowed assessing the exact position of the thermal spot and the overall safety profile of the applied sonication parameters. High-power sonications were then applied in an iterative process guided by MR imaging and MR thermometry, with stepwise increases in the acoustic power and energy to finally achieve a temperature at the target (9 central MR voxels) between 52°C and 59°C (average 56.2°C), the threshold for 100% necrosis being 54°C. The mean sonication duration was 13 s (range 10–21 s), the maximum acoustic power 1,200 W, and the maximum applied energy 20,400 J. The patients were fully awake and responsive during all the stages of the intervention. The only medication administered before the procedure consisted of sublingual lorazepam 1.25–2.5 mg and a gastric protection (pantoprazole 40 mg per os). During the entire series of sonications, the patients were examined and questioned repeatedly to ensure their neurological integrity. The pallidothalamic tractotomy (PTT) was performed unilaterally; there were nine right-sided and four left-sided PTT.

### MR examinations

The following series of MR sequences was performed, before PTT as base-line, at 2 days and at 3 months after PTT:

1) sagittal, axial, and coronal T2w FRFSE,

2) Axial 3D T1w BRAVO

3) Axial 3D SWAN

4) Axial diffusion tensor imaging with isotropic images post-processing.

All axial planes were cut parallel to the intercommissural plane and the coronal plane perpendicular to it.

### Target reconstruction method

The target reconstruction method has been described before [17]. In summary, the desired target coordinates from the Morel atlas were compared with the realized target geometric center coordinates as seen on sagittal and axial T2w series 2 days after PTT to obtain a global accuracy. The results are presented in the form of accuracy values for each of the three dimensions (mediolateral ML, anteroposterior AP, and dorsoventral DV) and as the 3D vector accuracy.

## Results

### Target precision

**Figure 2:**
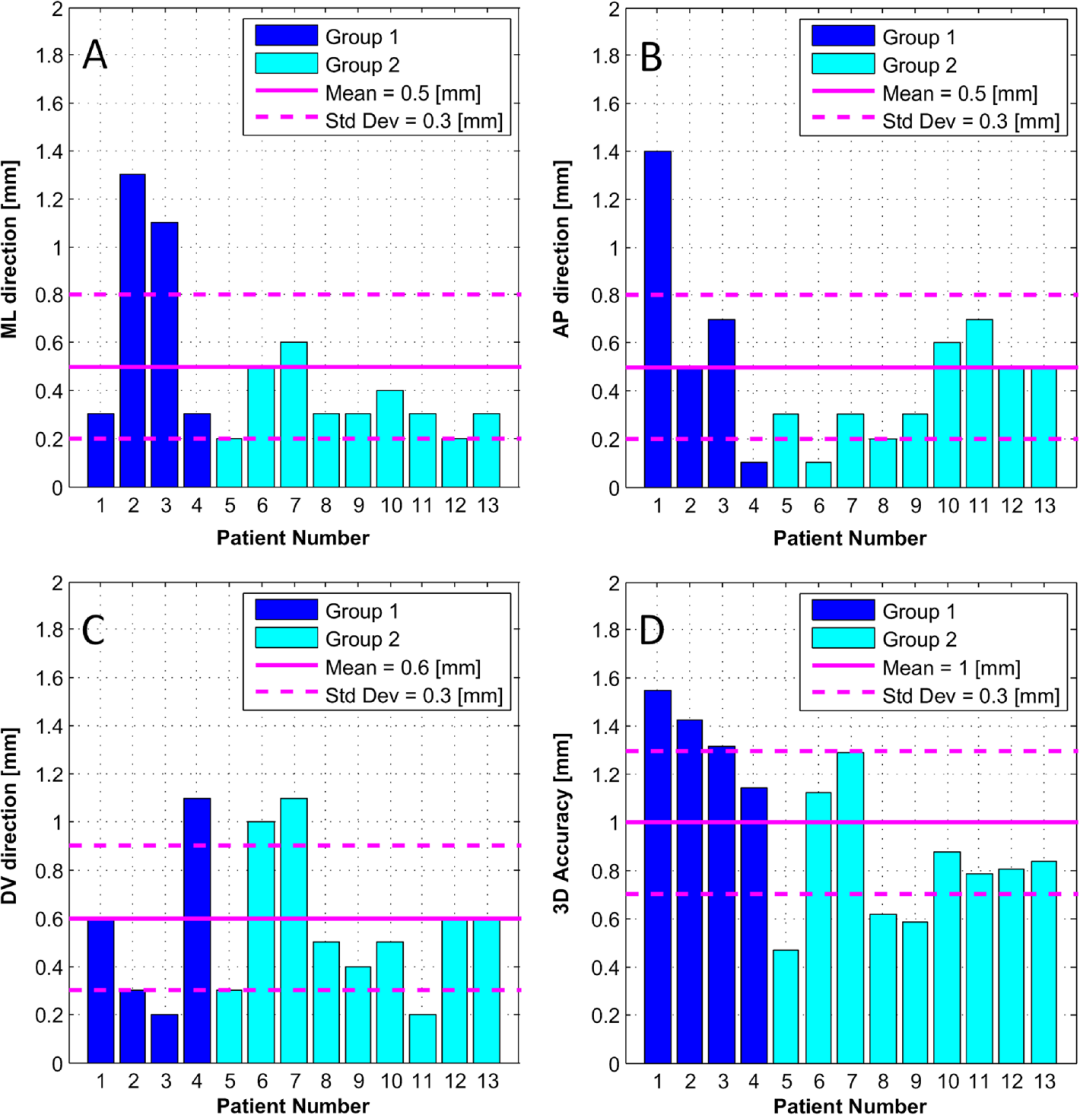
**Absolute global targeting accuracy.** The bar graphs display the absolute difference between the coordinates of the center of the atlas target and of the realized target (lesion) for the mediolateral (ML) direction **(A)**, anterioposterior (AP) direction, **(B)** and dorsoventral direction **(C)**, as well as the resulting three-dimensional vector length **(D)**. Std Dev stands for *standard deviation*.

**Figure 3:**
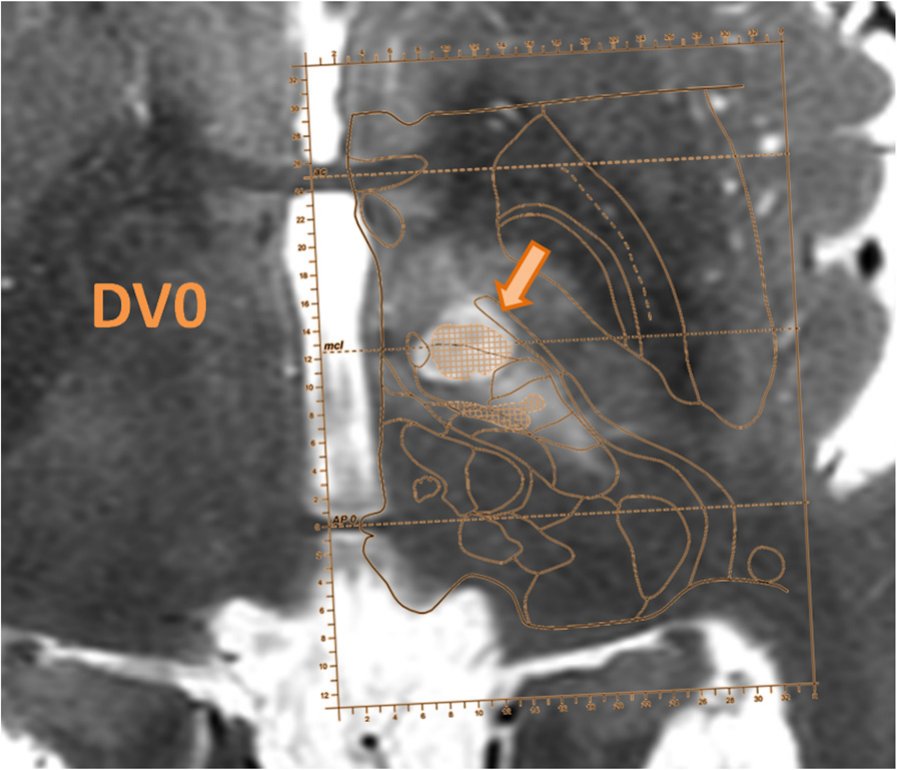
**Superposition of atlas map over MR imaging.** Axial MR T2-weighted (T2w) series taken at 2 days follow-up with superposition of the corresponding Morel atlas plane at the dorsoventral position 0 mm (DV0, the intercommissural plane). The hypodense lesion is exactly centered on the pallidothalamic tract as displayed on the atlas.

Figure 2 shows the targeting accuracy values of all performed lesions, with a mean absolute accuracy of 0.5 mm in the ML direction, 0.5 mm in the AP direction, and 0.6 mm in the DV direction. The mean 3D vector accuracy was 1.0 mm. Figure 3 shows an example of a PTT lesion on the intercommissural plane with superimposition of the pallidothalamic tract from the Morel atlas [18].

### Clinical data

**Figure 4:**
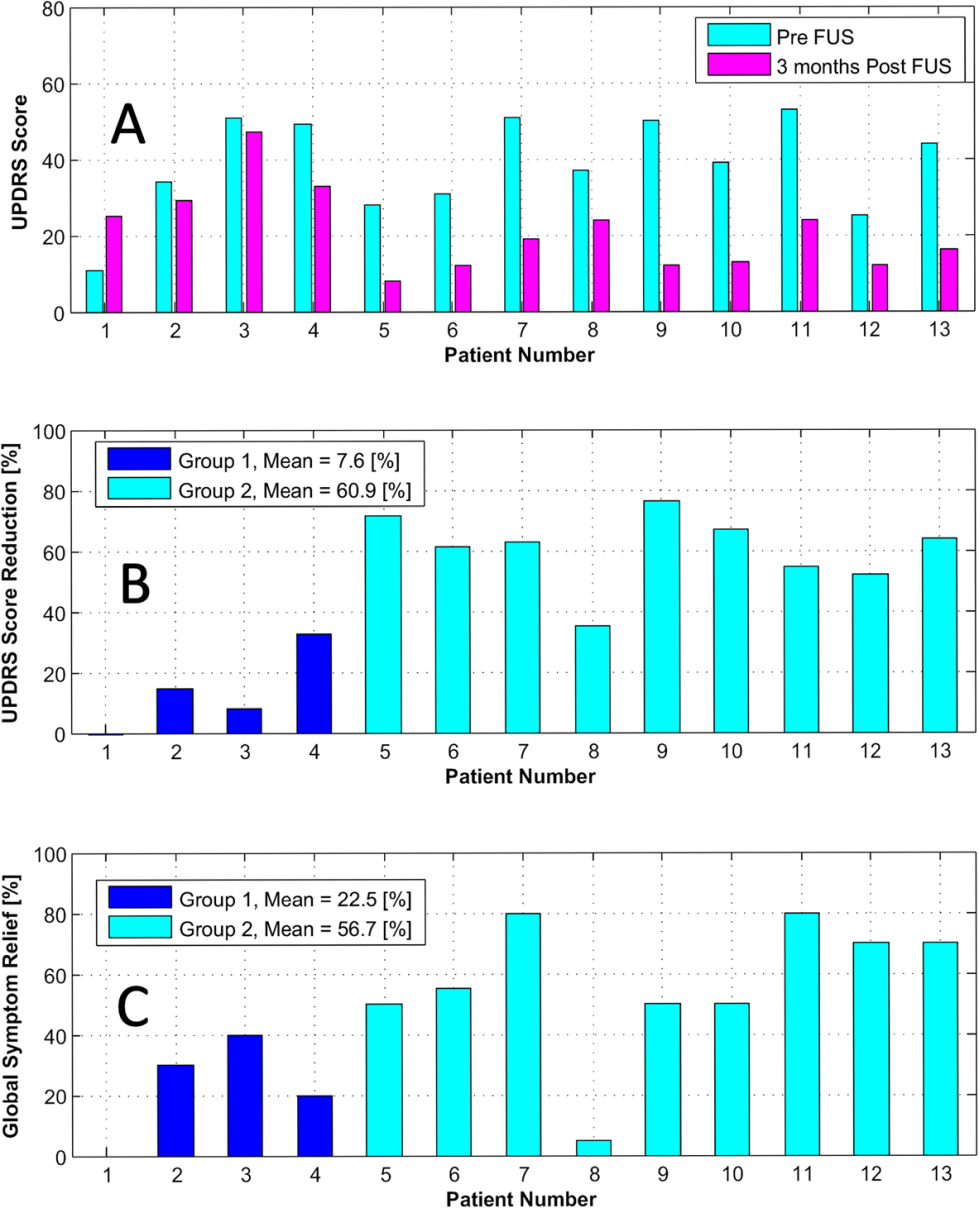
**UPDRS score and global symptom relief of the two groups. (A)** Pre- and 3 months post-FUS Unified Parkinson's Disease Rating Scale (UPDRS) score of the 13 patients. **(B)** UPDRS score reduction (in percent) for the same 13 patients. **(C)** Subjective Global Symptom Relief (3 months post vs. pre-FUS), in percent, for the 13 patients.

Table 2 displays the clinical results for the 13 consecutive PD patients. There were no procedure- or device-related neurological side effects. The final temperatures at target were averaged on the nine central voxels and ranged between 52°C and 59°C (average 56.2°C). The first four patients (group one) received on target a single application of the peak energy. For the next nine patients (group two), the peak energy application was repeated 4–5 times. The pre- and 3 months post-FUS UPDRS scores of the two groups ranged between 8 and 53, with a mean of 38.7 pre-FUS and 21.1 post-FUS (see Figure 4, top). Figure 4 shows on the mid-diagram the mean UPDRS score reduction for the first (7.6%) and for the second (60.9%) patient groups. The bottom diagram displays the GSR of the same two groups, 22.5% and 56.7%, respectively. The mean MMST and HADS scores were 29 and 14.1 before and 29.4 and 13 after the intervention, respectively (Table 2). Table 3 displays the pre- and postoperative medications. All patients received between 600–1,200 mg/day l-dopa equivalents before the intervention except two who had stopped it long before because of side effects and one who took only 300 mg/day to avoid them.

**Table 2:** Treatment characteristics

	**Patient no.**	**Sonication target and side**	**Final temperature (°C)**	**Global symptom relief 3 months (%)**	**Pre-FUS UPDRS I/II/III/IV (max. 16/52/56/23, total 147)**	**3 months post-FUS UPDRS I/II/III/IV (max. 16/52/56/23, total 147)**	**Pre-FUS MMST (max. 30)**	**3 months post-FUS MMST (max. 30)**	**Pre-FUS HADS (max. 42)**	**3 months post-FUS HADS (max. 42)**
Group 1	1	PTT right	52	0	0/2/3/6	2/11/5/7	29	30	17	20
					Total 11	Total 25				
	2	PTT right	52	30	0/14/17/3	0/10/17/2	30	30	9	4
					Total 34	Total 29				
	3	PTT right	57	40	0/18/29/4	1/20/24/2	29	29	12	18
					Total 51	Total 47				
	4	PTT right	59	20	0/18/26/5	0/12/20/1	27	27	13	9
					Total 49	Total 33				
Group 2	5	PTT right	59	50	0/5/18/5	0/1/7/0	30	30	5	9
					Total 28	Total 8				
	6	PTT left	57	55	1/10/15/5	0/6/6/0	29	30	20	19
					Total 31	Total 12				
	7	PTT left	57	80	4/21/20/6	1/12/2/4	30	30	13	14
					Total 51	Total 19				
	8	PTT right	58	5	0/10/22/5	0/9/15/0	29	30	11	9
					Total 37	Total 24				
	9	PTT left	57	50	4/17/19/10	1/5/2/4	30	29	34	26
					Total 50	Total 12				
	10	PTT right	56	50	3/9/20/7	3/6/4/0	29	30	14	18
					Total 39	Total 13				
	11	PTT right	56	80	0/19/29/4	2/9/13/0	30	29	12	3
					Total 53	Total 24				
	12	PTT right	56	70	0/5/14/6	1/3/8/0	29	30	9	10
					Total 25	Total 12				
	13	PTT left	54	70	3/22/11/8	3/4/9/0	26	28	14	10
					Total 44	Total 16				
Mean		**-**	56.2	**-**	**-**	**-**	29	29.4	14.1	13

The treatment characteristics include the target and side and the mean and maximum temperatures achieved during treatment. The subjective 3-month follow-up global symptom relief, as well as the pre- and post-FUS UPDRS scores shown here, are also displayed on Figure 4. The pre- and post-operative Mini Mental State Test (MMST) scores as well as Hospital Anxiety and Depression Scores (HADS) on the last four rows show no cognition losses and no emotional worsening for both groups of patients.

**Table 3:** Patient medication

	**Patient no.**	**Pre-FUS l-dopa equivalents (mg/day)**	**3 months post-FUS l-dopa equivalents (mg/day)**	**Pre-FUS dopamine agonists (mg/day)**	**3 months post-FUS dopamine agonists (mg/day)**
Group 1	1	1,000	1,000	Ropinirol: 3	Ropinirol: 3
				Amantadine: 300	Amantadine: 300
				Biperiden: 4	Biperiden: 4
	2	800	800	-	-
	3	300	-	-	-
	4	600	600	Biperiden: 6	-
Group 2	5	1,000	-	-	-
	6	1,000	-	-	-
	7	1,200	600	Ropinirol: 2	Ropinirol: 2
	8	-	-	-	-
	9	600	1,000	Rotigotine: 8	-
	10	1,000	600	Ropinirol: 10	-
	11	-	-	Amantadine: 1,000	Amantadine: 600
	12	800	800	Ropinirol: 4	Ropinirol: 4
	13	800	500	Amantadine: 300	Amantadine: 300
				Entacapone: 1,600	Entacapone: 1,000
Mean		827	536		

Pre- and postoperative antiparkinsonian medication. Patients 8 and 11 had stopped their l-dopa intake long before the intervention. Mean intakes of l-dopa equivalents were calculated over 11 patients.

### Magnetic resonance data

**Figure 5:**
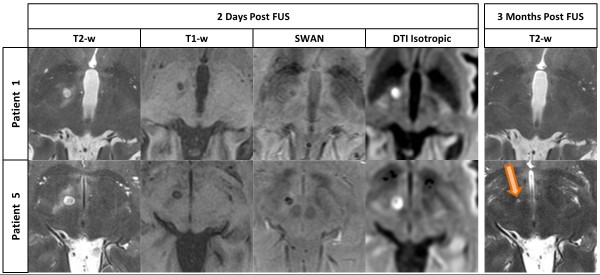
**MR imaging differences.** Lesion comparison between patient number 1 (group 1) and patient number 5 (group 2) on axial T2-weighted (T2-w), three-dimensional T1-weighted (T1-w), three-dimensional SWAN and Diffusion Tensor Imaging with isotropic post-processing MR series on 2 days and 3 months (only T2-w series) post-operative follow-up. Lesion can be seen on 3 months post-FUS MR imaging only in patient of group 2 (bottom, right). All scans are located 2 mm below the intercommissural plane.

Figure 5 shows, for two selected patients of the series, the T2w, T1w, SWAN, and DTI MR imaging at 2 days and 3 months. The first line displays the images of a patient of the first group, with good visualization at 2 days of the therapeutic lesion, lesion that became invisible at 3 months follow-up on the sensitive T2w imaging. The second line of the figure shows, in a patient from the second group, a lesion of larger volume at 2 days and its maintained T2w visualization at 3 months. Volume analysis of lesions using ellipsoid approximation method [17] showed larger lesion volumes for the second group (average of 172 mm^3^ against 83 mm^3^ for the first group). There were no target hemorrhages or other untoward tissue reactions.

## Discussion

To treat parkinsonian patients, neurosurgical procedures have been performed already in the 1960s [7-9] in the subthalamus. Like for pallidotomy, it was shown then and more recently that—in addition to tremor—akinetic and rigid symptoms could also be relieved, whereas thalamotomies only obtained a selective tremor relief [10]. Aufenberg et al. could demonstrate that RF PTT provided an improvement between 51% and 60% (as compared with the best medicated state) for the UPDRS II and III scores as well as the quality of life. There were different relief percentages for the various symptoms, and the only symptom which was pejorated was dysarthria (mean post-operative subscale 1/4, range 0–2/4). The results were stable over 5 years, and 21 over 41 patients could stop their drug intake. These results are better than what can be reached by HFS of the subthalamic nucleus, and there were no medium- or long-term problems as may be seen with HFS.

The MRgFUS technique allows performing stereotactic deep thermal ablations with reduced risks thanks to the absence of penetration through skin, bone, and brain and thanks to the on-line monitoring of the thermocoagulation process. In addition, the accuracy of the MRgFUS has been shown to be inside the millimeter, which is not obtainable with techniques implying brain penetration [17]. The results presented here confirm the overall post-operative reduction of the UPDRS score obtained with the RF PTT. There were no device- and procedure-related neurological side effects and no development of clinically relevant cognitive impairments.

Regarding thermal ablation, the radiofrequency tissue heating begins at the active surface of the electrode, the temperature distribution curve is relatively sharp and necessitates high peak temperature (at or above 80°C) to cover the desired lesion diameter (around 4 mm). In addition, the electrode produces a mechanical lesioning of the tissue above and in the target. The MRgFUS to the contrary causes exclusively a thermal lesion and covers the 4-mm diameter of the target with temperatures between 55°C and 60°C. Working with a less steep temperature distribution curve at or below 60°C produces a thermal lesioning which affects dominantly if not exclusively the neural tissue components. Indeed, lesioning at temperatures between 60°C and 70°C has been shown to cause capillary dilatation inside a soft necrosis, a situation representing an increased risk for bleeding [19]. Higher temperatures produced a hard necrosis of all tissue components. Before the experience described in the present study, we had no evidence of the relevance of differential thermal sensitivity of grey matter (nuclei) and white matter (fiber tracts). The clinical evidence has been collected, in accordance with earlier experimental data, that a single application of a peak temperature between 55°C and 60°C is enough to produce a complete nuclear thermocoagulation [16]. The PTT is however centered on a fiber bundle connecting the pallidum to the thalamus. Densely packed axons, protected by their myelin sheaths, may indeed need a stronger thermal application than a loose nuclear target, composed of cell bodies, astrocytes, and extracellular matrix. The clinical recurrences observed at 3 months in our first four patients gave a clear indication that the lesioning had been insufficient, which was confirmed by MR analyses. The final temperature expositions were able to avoid such recurrences and bring the results of the MRgFUS PTT to the level of the ones of the RF PTT (51% to 60% versus 60.9% for MRgFUS). It is to be noted that these repeated applications did not cause any untoward tissue reactions, including hemorrhages.

## Conclusions

The MRgFUS PTT provided similar clinical improvements to parkinsonian patients as the ones obtained by RF PTT. There were no post-operative neurological side effects. We were lead to recognize that repeated thermal applications were necessary for targets made of dense white matter. The RF technique could not provide this evidence, because of the higher temperatures involved and the addition of a mechanical lesion by the electrode.

## Competing interests

This research was supported partially by InSightec Ltd. (Haifa, Israel), Rodiag Diagnostics Centers AG (Olten, Switzerland), and GE Medical Systems (Switzerland). InSightec Ltd. is the manufacturer of the *ExAblate Neuro* System used in the study.

## Authors' contributions

AM, RB, and MK contributed to the conception and design of the study, carried out the pre- and postoperative clinical examinations, and revised the manuscript. DM contributed to the conception and design of the study, carried out the analysis of the targeting accuracy and MR data, and co-drafted the manuscript. PP contributed to the analysis and interpretation of the MR imaging data. DJ contributed to the conception and design of the study; the acquisition, analysis, and interpretation of the data; and co-drafted the manuscript. All authors read and approved the final manuscript.
